# Rapid multiplex MinION nanopore sequencing workflow for Influenza A viruses

**DOI:** 10.1186/s12879-020-05367-y

**Published:** 2020-09-03

**Authors:** Jacqueline King, Timm Harder, Martin Beer, Anne Pohlmann

**Affiliations:** Institute of Diagnostic Virology, Friedrich-Loeffler-Institut, Südufer 10, 17493 Greifswald, Insel Riems Germany

**Keywords:** Nanopore sequencing, MinION, Influenza A viruses, Full genome sequencing, Next-generation sequencing, Avian influenza viruses, Multiplexing

## Abstract

**Background:**

Due to the frequent reassortment and zoonotic potential of influenza A viruses, rapid gain of sequence information is crucial. Alongside established next-generation sequencing protocols, the MinION sequencing device (Oxford Nanopore Technologies) has become a serious competitor for routine whole-genome sequencing. Here, we established a novel, rapid and high-throughput MinION multiplexing workflow based on a universal RT-PCR.

**Methods:**

Twelve representative influenza A virus samples of multiple subtypes were universally amplified in a one-step RT-PCR and subsequently sequenced on the MinION instrument in conjunction with a barcoding library preparation kit from the rapid family and the MinIT performing live base-calling. The identical PCR products were sequenced on an IonTorrent platform and, after final consensus assembly, all data was compared for validation. To prove the practicability of the MinION-MinIT method in human and veterinary diagnostics, we sequenced recent and historical influenza strains for further benchmarking.

**Results:**

The MinION-MinIT combination generated over two million reads for twelve samples in a six-hour sequencing run, from which a total of 72% classified as quality screened, trimmed and mapped influenza reads to produce full genome sequences. Identities between the datasets of > 99.9% were achieved, with 100% coverage of all segments alongside a sufficient confidence and 4492fold mean depth. From RNA extraction to finished sequences, only 14 h were required.

**Conclusions:**

Overall, we developed and validated a novel and rapid multiplex workflow for influenza A virus sequencing. This protocol suits both clinical and academic settings, aiding in real time diagnostics and passive surveillance.

## Background

Next-generation sequencing (NGS) methods, especially second-generation sequencers, have shown their capability of whole-genome sequencing (WGS) over the past decade for a wide spectrum of pathogens including influenza A viruses (IAV) [1, 2]. Due to widespread avian influenza virus (AIV) outbreaks with high mortality among poultry and wild birds in combination with the unceasing risk of zoonosis, avian origin IAV has devastating economic and anthropological impacts [3, 4]. Frequent reassortment events and vast genetic diversity of these viruses show the necessity for fast and accurate WGS [5].

While the characterisation of IAV has greatly benefitted from WGS utilising first- and second-generation sequencers, limiting factors such as high costs, process duration, extensive protocols and large, stationary equipment leave room for improvement [6]. In recent times, the new era of third-generation sequencers has started to fill this gap. Among these, the portable MinION third-generation nanopore sequencing device (Oxford Nanopore Technologies, Oxford, UK; ONT) has developed to become a serious competitor [7], especially in regard to real-time sequencing and multiplex barcoding possibilities [8].

Employing the MinION with a broad range of IAV subtypes of both avian and human origin, we developed and validated a high-throughput sequencing workflow and speedy screening method for unknown IAV samples. During an outbreak situation, this method could dramatically reduce the cost and time for WGS, thus accelerating the response and aiding in disease control.

## Methods

### Nucleic acid extraction

Twelve egg-grown avian virus isolates (Table 1), four human IAV isolates and two avian swab samples (Additional File 1, Table S1) of different subtypes were collected in the German National Reference Laboratory for Avian Influenza, located at the Friedrich-Loeffler-Institut, Insel Riems, Germany. RNA was extracted using TRIZOL LS (Thermo Fisher Scientific, Waltham, USA) and the QIAamp Viral RNA Mini Kit (Qiagen, Hilden, Germany) according to the manufacturer’s instructions.

**Table 1:** Summary of MinION data from all reference samples sequenced with name, subtype and barcode. Mean coverage, mapped reads and number of nucleotide differences are shown for each individual segment. The consensus identity is calculated for all segments in %

Barcode	Sample	Subtype		PB2	PB1	PA	HA	NA	NP	MP	NS	Sum	Consensus identity %
1	R30–06	H1N1	**Mean Coverage**	749.9	188.3	700.9	2771.9	8756.7	7987.3	13,551.5	11,637.7	5793.025	99.98528113
			**Mapped Reads**	8102	1131	4059	8207	22,156	20,995	31,635	30,095	126,380	
			**Nucleotide Differences**				1	1				2	
2	R3111–07	H2N9	**Mean Coverage**	2250.2	1360.5	1001	4396.1	626.7	4582.6	8212.6	7477	3738.3375	99.99264057
			**Mapped Reads**	32,171	11,283	7372	11,560	2390	10,367	18,613	18,298	112,054	
			**Nucleotide Differences**				1					1	
3	R2555–06	H3N1	**Mean Coverage**	6373.3	2200.9	2080.6	929.1	1968	3874.8	5749.1	5016.4	3524.025	99.98528113
			**Mapped Reads**	40,737	14,924	14,166	4074	6248	9628	13,859	8970	112,606	
			**Nucleotide Differences**		1			1				2	
4	HAIV-81	H4N6	**Mean Coverage**	1462	3401.4	792.2	4161.2	8039.7	11,356.3	14,790.1	9955.9	6744.85	99.98528113
			**Mapped Reads**	18,920	20,238	6874	11,778	20,138	27,900	35,597	23,680	165,125	
			**Nucleotide Differences**	1			1					2	
5	R1612–08	H5N3	**Mean Coverage**	5033.4	3013.7	5260.7	1441.3	3776.2	1802.1	6314.7	5898.6	4067.5875	99.96320283
			**Mapped Reads**	35,383	19,354	31,659	8876	10,321	4821	14,220	12,908	137,542	
			**Nucleotide Differences**	1	1		2	1				5	
6	R617–07	H6N2	**Mean Coverage**	3933.5	2889.7	2995.6	4024	3403.6	3564.3	9820.9	5480.4	4514	99.99264057
			**Mapped Reads**	29,256	21,688	23,359	13,971	9179	8292	22,581	13,515	141,841	
			**Nucleotide Differences**				1					1	
7	R11–01	H7N7	**Mean Coverage**	5558.2	1201	1696.6	3737.4	3467.6	6623.9	7390.9	6832.7	4563.5375	99.98528113
			**Mapped Reads**	46,729	12,888	17,912	15,132	8502	16,267	18,781	16,333	152,544	
			**Nucleotide Differences**		1	1						2	
8	R249–08	H9N2	**Mean Coverage**	4445.5	3087.6	2260.4	1729.2	2652.5	2341.6	5403.8	6044.2	3495.6	99.98528113
			**Mapped Reads**	32,149	20,998	14,076	6316	7275	6366	12,914	13,244	113,338	
			**Nucleotide Differences**			1	1					2	
9	WV1677–03	H10N4	**Mean Coverage**	4296.7	1296.4	2896.6	3175.4	2728	3976.2	7689.3	5656	3964.325	99.9779217
			**Mapped Reads**	41,559	12,764	26,781	9445	8429	10,422	17,043	13,420	139,863	
			**Nucleotide Differences**			2		1				3	
10	R2675–06	H11N6	**Mean Coverage**	924.2	912.9	1378.1	1499.1	2260.8	4951.9	4345.1	3944	2527.0125	99.99264057
			**Mapped Reads**	14,361	12,470	13,834	5702	5402	12,233	10,004	9893	83,899	
			**Nucleotide Differences**	1								1	
11	R2613–06	H13N8	**Mean Coverage**	866.9	1040.9	1199.3	6152	5035.8	10,790.8	19,984.4	16,654.6	7715.5875	99.9779217
			**Mapped Reads**	9250	10,656	10,600	19,030	14,503	26,581	48,937	44,445	184,002	
			**Nucleotide Differences**	1	1			1				3	
12	Se-99	H16N3	**Mean Coverage**	9922	426.7	599.1	115.8	2673.5	2096	5058.4	5190.9	3260.3	99.96320283
			**Mapped Reads**	3843	2695	5041	393	11,992	5296	12,593	11,951	53,804	
			**Nucleotide Differences**	1	1		1	2				5	

### IAV-End-RT-PCR and purification

RNA was amplified with one pair of influenza-specific primers (forward and reverse) at a 10 pmol/μl concentration [9] using Invitrogen Superscript III One-Step RT-PCR with Platinum Taq (Thermo Fisher Scientific). The IAV-End-RT-PCR included 5 μl RNA template, 1 μl forward and 1 μl reverse primer, 12.5 μl reaction mix, 1 μl SuperScript III RT/Platinum Taq mix and 4.5 μl RNase free water to obtain a total volume of 25 μl. In this protocol, all influenza segments are amplified simultaneously using a one-step RT-PCR and one set of primers adapted to the conserved 3′ and 5′ segment ends. Amplicon length therefore ranges from the smallest non-structural protein segment (866 nt) to the largest polybasic 2 protein segment (2316 nt), in accordance to the individual segment lengths.

Cycling conditions for the respective IAV-End-RT-PCR were conducted as described: An initial primary reverse transcription step of 30 min at 55 °C, then denaturation at 94 °C for 2 min, followed by five cycles of 94 °C for 30 s, 45 °C for 30 s and 68 °C for 3 min, then an additional 30 cycles of 94 °C for 30 s, 57 °C for 30 s and 68 °C for 3 min, and to conclude a final elongation step at 68 °C for 5 min.

After amplification, samples were purified with AMPure XP Magnetic Beads (Beckman Coulter, Fullerton, USA) in an × 0.65 sample volume to bead volume ratio. Quantification was conducted with the NanoDrop™ 1000 Spectrophotometer (Thermo Fisher Scientific).

### Sequencing of IAV-End-PCR products – IonTorrent platform

The purified avian RT-PCR amplicons were sequenced on the IonTorrent platform (Thermo Fisher Scientific) as previously described [5, 10]. Before library preparation for the respective platform, the samples were mechanically fragmented to a 500 bp size on a Covaris M220 Ultrasonicator (Covaris Ltd., Brighton, UK). The GeneRead DNA L Core Kit (Qiagen) was subsequently used for library preparation with Xpress Barcode Adapters (Qiagen). After a following size selection and clean-up step with AMPure XP Beads (Beckman Coulter), the final library was quality checked on an Agilent Bioanalyzer 2100 (Agilent Technologies, Böblingen, Germany) and quantized via qPCR with the KAPA Library Quantification Kit (Roche, Mannheim, Germany). Sequencing was conducted on the IonTorrent S5XL (Thermo Fisher Scientific) in combination with the Ion OneTouch 2 System (Thermo Fisher Scientific), encompassing twelve AIV samples per Ion 530 Chip (Thermo Fisher Scientific).

### Analysis of IonTorrent sequencing data

The raw data produced was screened for adapter and primer contamination, followed by a quality trimming step. By using the Geneious Software Suite (v11.1.5; Biomatters, Auckland, New Zealand), consensus sequences were generated via a map to reference approach utilising Bowtie2 (v2.3.0; pre-set “Medium Sensitivity”) [11].

### Sequencing of IAV-End-PCR products – MinION sequencer

The identical purified IAV RT-PCR amplicons utilised on the IonTorrent were likewise employed for MinION sequencing along with the human strains. Following the manufacturer’s instructions, the Rapid Barcoding Kit (RBK-004, ONT) was applied: This 15 min two-step method includes a transposase for simultaneous cleaving of template DNA in conjunction with attachment of twelve barcodes to the cleaved ends (step 1), followed by pooling of the barcoded samples in the desired ratio and addition of Rapid Sequencing Adapters (step 2; ONT). After library preparation, the pooled samples were loaded onto a FLO-MIN106 R9.4.1 flow cell following the manufacturer’s instructions (ONT). A six-hour run was conducted with standard settings.

### Analysis of MinION sequencing data

Real time basecalling was performed with the MinIT and integrated Guppy v3.0.4 software (ONT) to produce fast5 and fastQ files. The automatic real time division into passed and failed reads by the MinIT works as a quality check, removing reads with quality scores < 7. The quality checked reads were demultiplexed and trimmed for adapters and primers using ONT Guppy Barcoding Software v3.1.5 + 781ed575, followed by mappings and a final consensus production in Geneious (v11.1.5; Biomatters) with Bowtie2 (v2.3.0; pre-set “Medium Sensitivity”) [11]. Due to the segmented influenza genome and thus comparable length of ONT and IonTorrent reads, usage of an identical mapping process was possible. MinION data quality was documented with NanoPlot v1.25.0 [12].

### Data availability

All sequence data (raw data, assemblies, consensus sequences) were made publicly available in the European Nucleotide Archive (ENA) under project accession PRJEB35098. Data accessions are summarised in Additional File 1, Table S2.

## Results

**Fig. 1:**
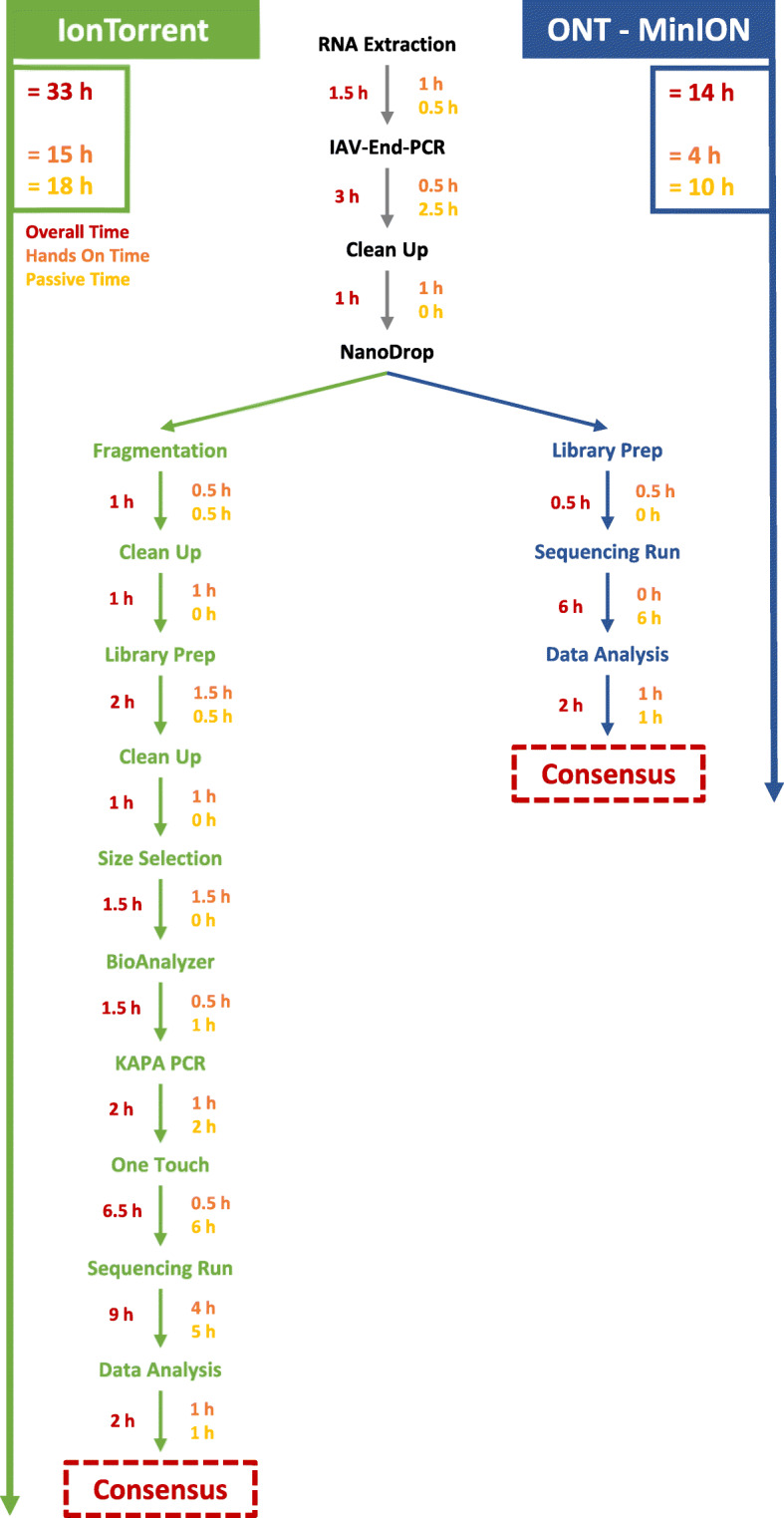
Comparison of IonTorrent S5XL and MinION workflow from RNA extraction to finished consensus sequences for twelve samples. Overall time is split into hands on and passive time needed for each protocol step

The IAV-End-RT-PCR successfully amplified all samples and segments. In the case of the IonTorrent run, 33 h were necessary to achieve twelve full genomes with 100% coverage (Fig. 1). A sum of 11,499,351 reads were produced after quality check and removal of polyclonal reads with an average of 97% classified influenza reads, evenly distributed between the individual barcodes. Overall, 258,488 reads were without a barcode (2.2%). An average trimmed read length of 276 nt was achieved.

For MinION sequencing, starting at the RNA extraction to final consensus sequence, a total of 14 h was necessary to produce twelve complete genomes with 100% coverage (Fig. 1). In total, 2,090,778 reads were generated in the six-hour run, of which 90.43% classified as passed reads. After demultiplexing, a final count of 1,667,946 reads (79.78% of overall reads, 87.01% of passed reads) was available for further analysis leaving 248,963 passed reads (11.91% of overall reads, 12.98% of passed reads) without or unable to be allocated to a barcode. The read distribution between the twelve barcodes was roughly even, with barcodes 10 and 12 producing less than average sequence counts (Additional File 1, Table S1). Read quality was monitored by evaluating read length versus read quality per sample (Additional File 1, Figure S1) with an overall mean read quality reaching from 12.3 to 12.6, typical for MinION data. Read length ranges from 375 to 567 nt with an average of 479 nt, making the data accessible for standard mapping algorithms.

Analyses of the generated full genome sequences revealed 1,522,998 mapped MinION reads (91.30% of all passed and demultiplexed reads) and a consensus identity between the IonTorrent and MinION data of > 99.9%, with a range of one to a maximum of five nucleotide differences throughout the entire genome. Respectively, virtually all deviations were detected in homopolymer regions. Altogether, a mean coverage of 4492 reads was achieved for all MinION data (Table 1).

The additionally MinION sequenced human IAV isolates and avian swab samples passed all quality checks to produce six further complete genomes with similar read quality and high coverage. The quality check process included the primary distinction of passed (quality scores > 7) and failed reads (quality scores < 7) during live basecalling and further evaluation of the read quality by conducting NanoPlot (v1.25.0) to receive, inter alia, mean read quality and read length. Here, the cut-off value for mean read quality was set at ≥10 for further usage of the sequencing data.

## Discussion

The proposed MinION workflow allows high-throughput sequencing in real time with a rapid library preparation protocol. Although MinION sequencing of IAV has previously been conducted [13], the novel combination of the IAV-End-RT-PCR with the Rapid Barcoding Kit (ONT) reduces the time for library preparation to a minimum and the analysis of low yield samples is achievable thanks to prior universal amplification. The respective RT-PCR allows for the production of less overall sequencing data due to the high proportion of viral influenza reads and a minimal host share, concurrently saving time and monetary means. The Rapid Barcoding Kit (ONT) also dramatically cuts the hands-on time needed for other NGS platforms, additionally reducing labour amount and, thus, expenditure. By utilising the MinIT (ONT), real time basecalling allows real time analysis, consequently leading to fast results, often crucial in the clinical setting.

Next-generation sequencers can produce immense amounts of data at a moderate cost, yet the application in clinical diagnostics is limited due to capital investment, complexity and time-consuming protocols. Easy, rapid and cost-efficient sequencing on the MinION platform could make sequencing accessible to a wide range of research backgrounds and might change the diagnostic process in the healthcare system. The availability of third-generation sequencers is pushing sequencing in the direction of becoming an integral part of many laboratories. The proposed IAV MinION sequencing protocol could easily be introduced into existing laboratory environments and allow direct, rapid and cost-efficient identification of diverse IAV strains. In addition, this protocol allows high-throughput sequencing of, for example, AIV samples from passive surveillance studies, shown to be of great importance for the molecular epidemiology of the worldwide AIV situation [14, 15].

The suitability of sequencing approaches for field surveillance was demonstrated with swine influenza viruses and the comparison to Illumina sequencing library strategies show comparable results with longer total analysis time and specific hands-on-time, respectively [16]. The use of multiplexing strategies is preferable due to the advantages of barcoding in higher throughput, better cost efficiency and decreased sequencing run time. The use of amplification with universal primers also allows an easier workflow and, in this study, allows better comparability of the achieved results, as the identical PCR products were sequenced on both platforms. PCR-free sequencing approaches for IAV have been described using direct RNA sequencing on the MinION platform to receive the complete coding genome of IAV [7]. This method allows avoidance of a prior PCR, and thus, the concurrent potentially resulting bias. Although this method is of great interest, the samples were high titre isolates and the limit of detection reached Ct values of 17, which lies outside the range of most clinical samples. Albeit the produced consensus sequences shared maximally 98.97% identity to the reference, the complexity and expenditure to achieve these results is not viable in the clinical setting. Multiplexing of direct RNA sequencing has yet to be produced for the MinION platform, additionally raising expenditures. In comparison, the here described MinION workflow allows superior consensus identity levels for low viral load samples at a lower cost and time consumption. In the future, direct RNA sequencing will certainly play a significant role; however, the current technological capabilities will most likely first allow the entry of third-generation sequencers into the clinical setting, in line with the aim of our proposed protocol.

Metagenomic nanopore sequencing has previously been piloted for clinical respiratory IAV samples [17]. The results of this study are promising for the combination of nanopore sequencing and metagenomics. However, although the detection of individual IAV reads was described in samples with Ct values of up to 36, whole genome sequences with the necessary coverage depth were only achieved at much lower Ct levels. Additionally, far larger datasets are needed to attain full genome coverage in comparison to the PCR-based MinION protocol. Deep sequencing and the concomitant possibility of SNP and variant detection is likewise only achievable with greater coverage depth, also attainable with the proposed protocol.

A multitude of varying bioinformatics analysis tools are available for sequencing data produced on ONT platforms, all aiming towards the improvement of the currently standing error profile. Especially in the current worldwide SARS-CoV-2 pandemic, third-generation sequencing platforms with distinct bioinformatic workflows have been implemented to obtain whole genomes [18, 19]. In the proposed protocol, the aim was to concentrate on the laboratory work and keep the implementation as accessible as possible, thus allowing better comparisons of both platforms via the utilisation of the identical annotation workflow. The comparability was shown by the generation of highly identical consensus sequences proving that the higher error rates of individual reads could be compensated by higher coverage.

The error rate of individual reads likewise affects demultiplexing of reads [20]. Misindexed reads are a known problem for nanopore sequencing, with on average 0.056% of total reads assigned to the incorrect barcode. When conducting metagenomic sequencing, a value of 0.056% misindexed reads can immensely influence the final genome construction, as often a fast majority of > 99% of the produced reads derive from the host [21, 22], leaving only few viral reads for analysis. Using the proposed method, high coverage and a large percent of viral influenza reads are expected due to the upstream IAV-End-RT-PCR (here: 91.3% of all passed and demultiplexed MinION reads were identified as influenza reads). Therefore, the fraction of misindexed reads does not affect the final consensus production and is, thus, in this case negligible.

Overall, sample processing of twelve samples can be achieved in 14 h, less than half the time required with the IonTorrent, without the need for large, expensive devices. Remarkable is the very low hands on time needed with the transposase-based rapid library MinION protocol. Although the accuracy of the MinION is known to be lower than other NGS platforms, especially struggling with homopolymer regions [23], adequate coverage leads to almost identical consensus sequences [24], as our data confirms. The MinION proved to be adaptably applicable for not only avian and human isolates, but also for representative clinical swab samples. In addition, swab samples from the recent 2020 outbreak of a novel IAV clade 2.3.4.4b H5N8 reassortant in Germany were successfully sequenced to produce full genome sequences using this method [25], demonstrating the practicality and applicability of the respective workflow.

Ongoing improvements from ONT are expected to advance basecalling accuracy with new technologies, e.g. the R10 flow cells, alongside the development of more accurate direct RNA sequencing kits to avoid PCRs and resulting biases. In addition, library preparation using automatic systems like the VolTRAX V2 (ONT) will allow even less hands-on time and reduce contamination while improving reproducibility.

## Conclusions

In summary, we developed and validated a novel rapid multiplex workflow for IAV sequencing using the MinION in combination with a one-step RT-PCR and the Rapid Barcoding Kit (ONT). This protocol is ideal for both clinical and academic settings, aiding in real time diagnostics, applicable to any IAV sample and indispensable for active outbreaks and passive surveillance.

## Supplementary information

**Additional file 1.**

## Data Availability

The datasets generated and analysed in the current study are available in the European Nucleotide Archive (ENA) under project accession PRJEB35098. Data accessions are summarised in Additional File 1, Table S2.

## Abbreviations

IAV: Influenza A viruses
AIV: Avian influenza viruses
HPAIV: Highly pathogenic avian influenza viruses
LPAIV: Low pathogenic avian influenza viruses
ONT: Oxford Nanopore Technologies
RT-PCR: Reverse transcription polymerase chain reaction
NGS: Next-generation sequencing
WGS: Whole-genome sequencing
ENA: European Nucleotide Archive
RNA: Ribonucleic acid
Nt: Nucleotide
