# Supplementary material for: Rapid multiplex MinION nanopore sequencing workflow for Influenza A viruses
Source: BMC Infect Dis. 2020 Sep 3;20:648. doi: 10.1186/s12879-020-05367-y (PMC7468549; doi:10.1186/s12879-020-05367-y)

Additional File 1 - Rapid Multiplex MinION Nanopore Sequencing Workflow for Influenza A Viruses

TABLE S1 Metrics of MinION and IonTorrent sequencing of twelve influenza reference samples, four human IAV samples and two avian IAV swab samples.

Sample information		ONT MinION					IonTorrent S5				
**Sample ID**	**Subtype**	**Barcode**	**No. reads**	**No. nucleotides**	**Mean read length**	**Mean read quality**	**Barcode**	**No. reads**	**No. nucleotides**	**Mean read length**	**Mean read quality**
**R30-06**	**H1N1**	RBK_01	135,319	76,779,158	567	12.4	IonXpress_001	979,254	263,435,070	269	27.5
**R3111-07**	**H2N9**	RBK_02	124,950	57,837,095	463	12.3	IonXpress_002	789,774	219,019,649	277	27.3
**R2555-06**	**H3N1**	RBK_03	127,224	47,715,460	375	12.4	IonXpress_005	896,250	249,270,903	278	26.8
**HAIV-81**	**H4N6**	RBK_04	184,108	97,179,395	528	12.4	IonXpress_006	945,428	255,182,523	269	27.2
**R1612-08**	**H5N3**	RBK_05	154,055	58,279,146	378	12.3	IonXpress_007	1,141,716	319,299,737	279	26.8
**R617-07**	**H6N2**	RBK_06	155,749	66,775,697	429	12.5	IonXpress_008	584,795	166,306,709	284	26.6
**R11-01**	**H7N7**	RBK_07	171,932	73,404,138	427	12.3	IonXpress_010	911,348	245,062,731	268	27.2
**R249-08**	**H9N2**	RBK_08	127,146	47,167,977	371	12.5	IonXpress_011	991,208	272,905,748	275	26.9
**WV1677-03**	**H10N4**	RBK_09	149,147	61,256,718	411	12.5	IonXpress_012	988,573	278,693,189	281	26.9
**R2675-06**	**H11N6**	RBK_10	89,162	40,107,611	450	12.6	IonXpress_013	940,860	261,449,754	277	27.1
**R2613-06**	**H13N8**	RBK_11	192,947	101,885,364	528	12.4	IonXpress_014	1,038,855	294,774,287	283	27.2
**Se-99**	**H16N3**	RBK_12	56,207	27,001,365	480	12.5	IonXpress_015	988,802	266,418,429	269	27.2
**FM-47**	**H1N1**	RBK_04	312,507	171,295,702	548	12.3	NA	NA	NA	NA	NA
**R1541-07**	**H1N1**	RBK_05	310,449	187,350,645	603	12.4	NA	NA	NA	NA	NA
**H1N1-swl**	**H1pdmN1**	RBK_06	308,631	197,777,138	640	12.2	NA	NA	NA	NA	NA
**AR3343-17**	**H1pdmN1**	RBK_07	390,993	234,193,266	599	12.5	NA	NA	NA	NA	NA
**AR780-17**	**H5N5**	RBK_10	15,402	9,615,096	624	12.5	IonXpress_089	426,862	127,641,921	299	27.3
**AR1384-17**	**H5N8**	RBK_11	79,149	48,446,910	612	12.3	IonXpress_093	426,540	134,969,178	316	27.0

TABLE S2 Accessions numbers (ENA) of shard data under project accession PRJEB35098.

Sample ID	Virus ID	Subtype	Sample Accession	Run Accession ONT	Run Accession Ion Torrent
R30-06	A/duck/Germany/R30/2006	H1N1	ERS4226848	ERR3822170	ERR3822188
R3111-07	A/duck/Germany/R3111/2007	H2N9	ERS4226849	ERR3822171	ERR3822189
R2555-06	A/duck/Germany/R2555/2006	H3N1	ERS4226850	ERR3822172	ERR3822190
HAIV-81	A/unknown/unknown/HAIV/1981	H4N6	ERS4226851	ERR3822173	ERR3822191
R1612-08	A/turkey/Germany//R1612/2008	H5N3	ERS4226852	ERR3822174	ERR3822192
R617-07	A/turkey/Germany/R617/2007	H6N2	ERS4226853	ERR3822175	ERR3822193
R11-01	A/turkey/Germany/R11/2007	H7N7	ERS4226854	ERR3822176	ERR3822194
R249-08	A/avian/Germany/R249/2008	H9N2	ERS4226855	ERR3822177	ERR3822195
WV1677-03	A/mallard/Germany/WV1677/2003	H10N4	ERS4226856	ERR3822178	ERR3822196
R2675-06	A/dunnock/Germany/R2675/2006	H11N6	ERS4226857	ERR3822179	ERR3822197
R2613-06	A/black-headed gull/Germany/R2613/2006	H13N8	ERS4226858	ERR3822180	ERR3822198
Se-99	A/black-headed gull/Sweden/5/1999	H16N3	ERS4226859	ERR3822181	ERR3822199
FM-47	A/Fort Mammoth/1/47	H1N1	ERS4226860	ERR3822182	NA
R1541-07	A/Niedersachsen/14/07	H1N1	ERS4226861	ERR3822183	NA
H1N1-swl	A/Regensburg/D6/09	H1pdmN1	ERS4226862	ERR3822184	NA
AR3343-17	A/Michigan/45/2015	H1pdmN1	ERS4226863	ERR3822185	NA
AR780-17	A/turkey/Germany/AR780/2017	H5N5	ERS4226864	ERR3822186	NA
AR1384-17	A/chicken/Germany/AR1384/2017	H5N8	ERS4226865	ERR3822187	NA

FIGURE S1 Read quality versus read length of MinION data using Nanoplot.

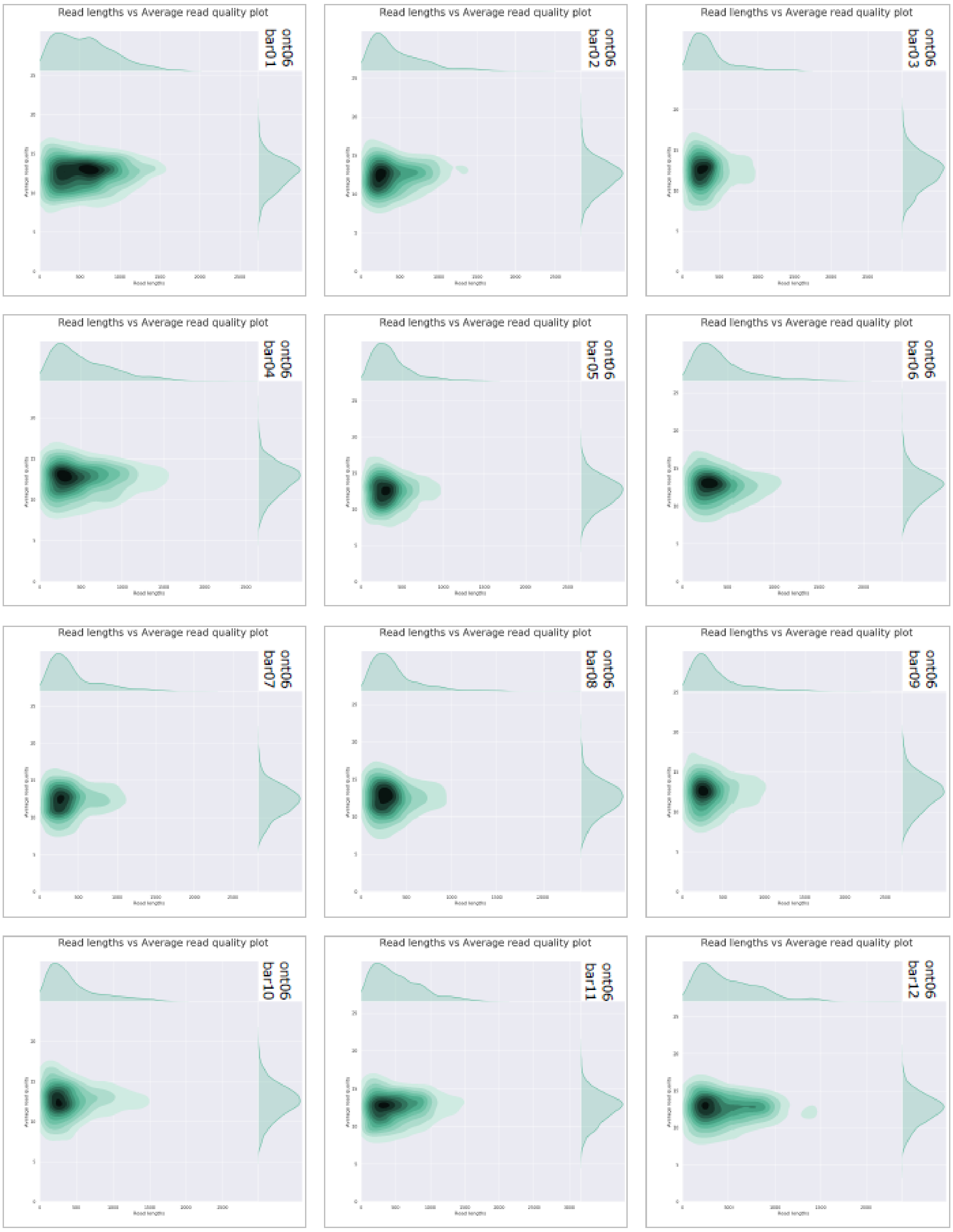

Supplement: Supplementary file 1 — Additional file 1. [file 12879_2020_5367_MOESM1_ESM.pdf]
